# A Historical Review on the Andalusian Physicians and the Treatment of Mental Health

**DOI:** 10.1007/s11673-024-10412-5

**Published:** 2025-04-25

**Authors:** H. Alotaibi

**Affiliations:** Imam Abdulrahman Bin Faisal University, Dammam, Saudi Arabia

**Keywords:** Mental health, Anxiety, Stress, Andalusia, Physicians, History

## Abstract

Andalusian medicine, characterized by its holistic approach to healthcare, placed a unique emphasis on the interconnectedness of physical health, emotional well-being, and spiritual harmony. Eminent scholars, including physicians like Ibn Sina, Ibn al-Nafis, and Ibn Wafid, pioneered advancements in understanding mental disorders, the complexities of the human psyche, and the intricate relationship between the body and mind. One of the most enduring legacies of Andalusian contributions to mental health was the establishment of mental health hospitals, known as “maristanes.” These institutions, distinguished by their compassionate and patient-centred care, profoundly influenced the development of Western medicine, and laid the groundwork for the emergence of psychology as a scientific discipline. This paper goes through the intellectual tapestry of Andalusia during the historical era of Islamic governance, commonly known as the Andalusian Renaissance. Situated in the heart of the Iberian Peninsula, this period of remarkable intellectual convergence and cultural exchange is celebrated for its profound contributions to the field of mental health.

## Introduction

Within the records of the past, there is a notable period that exhibits a remarkable display of intellectual prowess, wherein the relentless quest for knowledge and advancement flourished within a rich tapestry of diversity and the exchange of cultural ideas. The current paper considers a place within the central region of Al-Andalus, a notable area located in the Iberian Peninsula that experienced significant growth and development during the Islamic era, thus making a lasting impact on the course of human history. The Andalusian civilization is renowned for its significant contributions, with a particular emphasis on the field of medicine, namely in the domain of mental health (Ribeiro 2019; Said 2017).

The historical era of Islamic governance in Andalusia, which endured from around the eighth century to the fifteenth century, was characterized by the convergence of diverse cultures, ideas, and intellectual legacies. During a specific historical period, there was a convergence of the teachings from ancient Greece, the wisdom originating from Persia, and the innovations emerging from India, which intersected with the intellectual traditions upheld by Islamic academics (Menocal 2006; Makki 1992; Casewit 2017; Corfis 2009). The convergence of diverse knowledge fostered a conducive atmosphere for intellectual development and ground-breaking advancements, particularly in the realm of medicine, encompassing the comprehension and management of mental disorders.

The historical account of Andalusian medicine, particularly its treatment of mental health, holds significant normative and philosophical relevance beyond its historical context. Medicine and medical philosophy are inherently intertwined, and the Andalusian approach, with its holistic emphasis on the interconnectedness of body, mind, and spirit, provides critical insights into both the philosophy of mind and the philosophy of medicine. This history is not merely an academic exercise but offers profound reflections on the conception and definition of mental illness, therapeutic rationale, and care. Furthermore, in a field often dominated by Western and Christian perspectives on medical history and bioethics, the Islamic contributions of Andalusian physicians provide an essential counter-narrative. These contributions challenge and enrich contemporary discussions, offering a more comprehensive understanding of mental healthcare ethics. The maristanes, for instance, with their focus on compassionate, gender-sensitive, and humane treatment of individuals with mental illness, present an ethical model that contrasts sharply with the later asylum movement in Europe and Anglo-American medical traditions. Understanding these contributions is critical not only for historians but also for ethicists, philosophers, and modern healthcare practitioners who aim to contextualize and broaden the foundations of medical ethics and mental healthcare.

As we begin an exploration into the contributions made by physicians from Andalusia to the realm of mental health, we find ourselves transported to a realm where the examination of the human psyche and its ailments was not solely a medical undertaking but rather a profound pursuit including philosophy and humanitarianism. This review article aims to provide an analysis of the significant contributions made by researchers from the Andalusian region in the field of mental health. It explores their valuable insights into the comprehension of mental disorders, the advancement of treatment methodologies, and the founding of ground-breaking mental health hospitals.

## Historical Background of Andalusian Medicine

In order to fully comprehend the importance of Andalusian medicine, it is imperative to gain a comprehensive understanding of the historical circumstances that facilitated its flourishing. During a period characterized by intellectual stagnation and cultural decline in many parts of Europe, the region of Andalusia had a notable flourishing, emerging as a prominent centre of knowledge and cultural development. The flourishing observed during this period can be attributed to a noteworthy convergence of several cultures, encompassing the Indigenous Iberian, Visigothic, Jewish, Christian, and Islamic influences (Corfis 2009). The basis of Andalusian medicine was established on a foundation of extensive historical knowledge. The Islamic world revitalized and repurposed the classical Greek, Roman, Persian, and Indian traditions, drawing upon their wisdom and knowledge. The pursuit of knowledge within the Islamic tradition, as demonstrated by the Quran’s directive to “read,” had a significant role in fostering the intellectual enthusiasm of intellectuals in Andalusia (Alfonso 2007).

### The Andalusian Renaissance

The Andalusian Renaissance, commonly known as the “Golden Age” of Islamic Spain, was distinguished by a profound desire for intellectual enrichment, scientific exploration, and intercultural interchange. During this period, intellectuals of diverse backgrounds convened with the purpose of translating, synthesizing, and advancing the knowledge contained within the writings of their predecessors. A notable characteristic of Andalusian medicine was its dedication to the preservation and progression of ancient knowledge. The Greek medical writings authored by prominent figures such as Hippocrates and Galen underwent meticulous translation efforts into the Arabic language. The translations provided a fundamental basis for Andalusian physicians to develop their comprehension of medicine, anatomy, and pharmacology (Renima et al. 2016; Lowney 2005).

### Holistic Approach to Medicine

The distinguishing characteristic of Andalusian medicine was its comprehensive approach to healthcare. The concept of the interrelation between the body and mind was acknowledged by physicians in Andalusia, a notion that is currently obtaining more recognition within the field of contemporary medicine. The individuals comprehended the interconnectedness between physical health, emotional well-being, and spiritual harmony. The comprehensive viewpoint was not solely confined to theoretical contemplations but also manifested itself practically in the way patient care was approached. The physicians from Andalusia were renowned for their sensitive and caring approach towards patient care. The holistic approach to health employed by the individuals in question placed significant emphasis on preventive medicine, dietary advice, and even psychological counselling (El-Gammal 1990; El-Gharbaoui et al. 2017).

### The Impact and Significance of Legacy

The impact of Andalusian medicine transcends the geographical confines of Al-Andalus. The knowledge and inventions formulated by experts from the region of Andalusia were widely transmitted across the Islamic world, exerting a significant influence on medical practices in distant locations such as Baghdad and Cairo (Tayarani-Najaran et al. 2014). Furthermore, the literary productions of Andalusian medical experts were rendered into Latin throughout the period of the European Renaissance, so making a substantial contribution to the resurgence of medical erudition in Europe. The impact of Andalusian medicine is clearly discernible in the advancement of the scientific method (Pormann and Savage-Smith 2007). The researchers of Andalusia, via the utilization of previous scholarly contributions and the implementation of empirical observations, established the fundamental principles for the methodical and evidence-driven methodology that characterizes contemporary medical practice.

## Understanding Mental Illness in Andalusia

During the medieval period in Europe, there existed a prevalent notion that specific ailments were attributed to divine curses. In contrast, Muslim theologians held the belief that disease served as a method of atonement. In contrast, the endeavour of addressing bodily symptoms was regarded as a commendable undertaking due to its ability to reinstate the overall health and wellness of patients. Academic scholars hailing from the lineage of Prophet Muhammad also actively participated in efforts aimed at altering societal perspectives on diseases. Jabir bin Hayyan (d.813) is widely recognized as a prominent figure in Arab chemistry and a key contributor to the development of modern pharmacy. He attributes his understanding of chemistry to Ja’far al-Sadiq (d.765). Musa al-Kazim, the son of Ja’far, who passed away in 799, would reiterate the teachings of the Prophet (Maravia and Al-Ghazal 2021), stating:

When a believer falls ill, Allah instructs the scribe to the patient’s left side: “Do not record a single sin against my servant as long as he is in my custody, and in my hold.” And He instructs the scribe to the right: “Write for my servant on his page of good deeds that which you would have recorded for him when he was well. (Al-Tabarsi 2004)

Muslim physicians throughout the Abbasid golden era drew inspiration from the teachings of the Prophet Muhammad and his heirs. A significant hadith asserts that there exists a divinely given remedy for every ailment. According to the source, individuals are advised to get medical treatment for their ailments (Ibn Abi Shaibah 1988). The transition towards addressing the spiritual and physical well-being of patients necessitated that medical practitioners had qualifications in medicine, rather than relying exclusively on theological expertise. According to historical records, Prophet Muhammad issued a cautionary statement to society, emphasizing the importance of holding individuals accountable if they were to engage in the practice of medicine without possessing the necessary qualifications (Al-Sijistani 2000). Consequently, bimaristans (Hospitals) only employed licenced physicians who were well regarded and remunerated generously.

## Andalusian Medical Scholars

The historical account of Andalusian medicine is abundant with renowned figures whose impact reverberates throughout the chronicles of medical history. One notable individual among them is Ibn Sina (Avicenna), a polymath originating from the region that is presently recognized as Uzbekistan. The “Canon of Medicine,” authored by him, emerged as a seminal contribution to medical pedagogy in Europe, exerting a lasting influence for numerous generations. Ibn al-Nafis, a prominent figure in the field of Andalusian medicine, made significant advancements in our comprehension of the circulatory system, predating the findings made by William Harvey in Europe by several centuries. Ibn al-Nafis presented a novel perspective in contrast to the dominant beliefs of his era, proposing that the heart serves as the origin of blood circulation, pumping it to the lungs for oxygenation prior to its distribution throughout the body. The contributions of his study have established the fundamental basis for our contemporary comprehension of the cardiovascular system (Ihsanoglu 2020; Al-Majali 2017).

Al-Zahrawi, alternatively referred to as Abulcasis in Latin, was an eminent physician and surgeon from Andalusia who played a pioneering role in advancing the discipline of surgery during the Islamic Golden Age. The literary contribution known as “Al-Tasrif” is widely recognized as a very influential and all-encompassing medical manuscript throughout its era. provided visual representations and detailed explanations about the utilization of diverse surgical instruments, encompassing scalpels, forceps, retractors, as well as specialized tools tailored for specific surgical interventions (Nouri-Vaskeh 2020).

Ibn Zuhr presented a precise delineation of esophageal, stomach, and mediastinal malignancies, along with additional pathological abnormalities. He suggested the administration of enemas as a means of sustaining individuals diagnosed with gastric cancer. Additionally, he was the pioneering physician to provide pathological descriptions of inflammatory conditions such as otitis media and pericarditis (van den Tweel and Taylor 2010; Weiss 2000).

Ibn Wafid, a distinguished physician hailing from Al-Andalus, made significant contributions to the domain of mental health. The author extensively explored a range of medical subjects, encompassing mental disorders among them. The author’s body of work encompassed detailed accounts of various psychological diseases, delineating their respective symptomatology, and exploring potential therapeutic interventions. The writings of Ibn Wafid exemplified the comprehensive perspective of Andalusian medicine, wherein the interdependence between mental health and physical well-being was emphasized. The primary contribution of the individual in question is in his notable publication titled Kitāb al-adwiya al-mufrada (كتاب الأدوية المفردة), which has been translated into Latin as De medicamentis simplicibus. Ibn al-Wafid primarily practiced pharmacy in Toledo, employing alchemical procedures and methodologies to derive a minimum of 520 distinct medicinal compounds from a diverse range of botanical sources. The renowned botanical dictionary, titled ʿUmdat al-Ṭabīb fī Maʿrifat al-Nabāt li kulli Labīb, was authored by Ali Ibn al-Lukuh, a notable student (Kaya 1999; Muñoz 1996; Emilia 1997).

Ibn Rushd is another renowned for his contributions to philosophy and jurisprudence, his philosophical writings often touched upon the human mind and consciousness. His works influenced not only philosophy but also early psychology. Ibn Rushd’s exploration of the mind’s functions and the relationship between the body and soul had a broader impact on the understanding of mental processes during his time (Virani 2011; Fakhry 2001; Glasner 2009).

Another prominent Andalusian philosopher and physician, Ibn Tufail, contributed to the understanding of the human mind. His philosophical novel, “Hayy ibn Yaqzan,” explored themes related to psychology, self-awareness, and the development of the human mind in isolation. While not a traditional medical text, his work delved into aspects of mental health and self-discovery (Amber 2004; Dominique 1996).

## Therapeutic Approaches for Mental Health in Andalusia

### Spiritual and Religious Guidance

Given the strong influence of Islamic culture and spirituality in Andalusia, some therapeutic approaches incorporated spiritual and religious guidance. Physicians often encouraged patients to engage in prayer, meditation, and spiritual reflection to find solace and emotional balance. While treatment was considered as the major approach for addressing mental health issues like anxiety, depression, stress etc., the spiritual approach of keeping faith in God was also considered. In order to address maladaptive cognitions associated with feelings of hopelessness and being overwhelmed by life, it is important to acknowledge that within the Muslim faith, there is no room for despair. This is because Muslims firmly believe in the omnipotent nature of God, who is characterized as possessing omniscience, fairness, and wisdom (Hamdan 2008).

And for those who fear Allah, He always prepares a way out, and He provides for him from sources he never could imagine. And if anyone puts his trust in Allah, sufficient is Allah for him. For Allah will surely accomplish His purpose: verily, for all things has Allah appointed a due proportion. (Quran, 65: 2–3)

Research conducted on individuals of the Muslim faith who underwent spiritually modified cognitive therapy for anxiety and depression demonstrated more expeditious outcomes as compared to therapy that did not incorporate Islamic principles. In a similar vein, a research investigation was undertaken to examine the effects of cognitive-behavioural therapy, tailored to integrate Islamic beliefs and practices, on Muslims experiencing depression. The findings of this study (Azhar and Varma 2000) revealed notable improvements in the outcomes of participants. A notable investigation was carried out on individuals diagnosed with schizophrenia who identified as Muslim in Saudi Arabia. The study demonstrated that religiously adjusted cognitive therapy yielded comparable or greater outcomes compared to those gained by conventional cognitive therapy (Wahass and Kent 1997).

### Aromatherapy

The use of aromatic substances, such as essential oils and fragrant herbs, was a common therapeutic approach. These aromas were believed to have a positive impact on mood and emotional well-being (Aguirre de Cárcer 2001).

### Herbal Remedies and Compounds

Andalusian physicians developed and prescribed specific herbal remedies and medicinal compounds for treating various mental health conditions. These treatments often combined herbs, spices, and other natural ingredients to address psychological symptoms (Ricordel 2008).

### Psychotherapy

While psychotherapy as we know it today was not developed in its modern form during the Andalusian period, physicians did engage in therapeutic conversations with patients. They offered counselling, empathy, and support to individuals with mental health issues, recognizing the importance of addressing psychological and emotional distress (Mitha 2020).

### Pharmacological Interventions

Andalusian physicians were pioneers in the use of pharmacological treatments for mental illnesses. They developed and prescribed herbal remedies, compounds, and elixirs to address various psychological disorders. These treatments aimed to restore the balance of bodily humours and alleviate symptoms (Casal and Casal 2004).

### Dietary Interventions

Nutrition was considered an essential aspect of mental well-being. Andalusian physicians recognized the influence of diet on mental health and often prescribed specific dietary regimens to patients with mental disorders. Dietary interventions aimed to correct imbalances in bodily humours and promote overall health (Inskip et al. 2019).

## The Establishment of Mental Health Hospitals in Andalusia

One of the most enduring legacies of Andalusian contributions to mental health was the establishment of mental health hospitals. These institutions, known as “maristanes,” were centers of care and compassion where individuals suffering from mental illnesses were treated with dignity and respect. The maristanes were not mere asylums; they were designed to provide holistic care, offering medical treatment, psychological support, and even recreational activities to promote healing. The architectural and organizational aspects of these mental health hospitals were revolutionary for their time. They were often designed with serene gardens and open spaces, recognizing the therapeutic value of nature and tranquility. Moreover, these hospitals employed a multidisciplinary approach to treatment, bringing together physicians, nurses, and counsellors to provide comprehensive care. Bimaristans were institutions that considered the need of both patients and their families. The demands encompassed various dimensions, including spiritual, mental, bodily, and social needs (Al-Ghazal 2007). Due to the high probability of patients having a religious affiliation, bimaristans incorporated designated prayer rooms specifically intended for the patients’ use. As an example, it can be observed that the Bimaristan Al-Mansuri in Cairo encompassed designated spaces for prayer, catering to adherents of the Islamic faith as well as those individuals who subscribed to the Abrahamic religions (Islam Message 2020). In addition to hospitals, areas of worship were incorporated into the infrastructure, ensuring that medical treatment was accessible to travellers en route to Makkah. The significance of the yearly Hajj pilgrimage is accompanied by the establishment of road bimaristans, which were funded by philanthropists (Ibn Kathir 1986).

The bimaristans also considered the patients’ social requirements. Muslim communities often place a high degree of importance on the provision of services that are tailored to specific genders. Moreover, it is important to note that bimaristans were equally essential in both rural areas and villages, in addition to their significance in urban centres. The Qur’an also promotes the display of benevolence towards convicts, which led to the provision of medical assistance to incarcerated individuals (The Holy Qur’an, chapter 76, verse 8). The bimaristans were organized into wards based on both disease type and gender. They hold significance within Islamic culture, as Muslim women exhibit a tendency to refrain from unnecessary exposure of their bodies, particularly in the presence of men who are not part of their immediate family. Nevertheless, due to the necessity of medical examinations involving the exposure of the abdomen or genitalia, it was deemed appropriate to establish distinct wards for men and women. In instances when feasible, women were attended to by female medical professionals. The provision of medical care to address these specific needs served as a catalyst for women to pursue careers in the field of medicine (Kump 1974).

The bimaristans also implemented measures to mitigate patients’ distress and concerns. Bimaristans were established in locations characterized by optimal air quality and scenic appeal. Bimaristans were also shown to have facilitated the return of health by ensuring that patients did not perceive themselves as abandoned to their fate. In addition, bimaristans were constructed within urban areas, allowing for accessibility to family and friends, who held the aspiration of reintegrating individuals into society after their recuperation (Miller 2006; Hamarneh 1962). Within the geographical region of the Iberian Peninsula, it is noteworthy that the city of Cordoba possessed a substantial number of fifty prominent hospitals. Certain medical facilities were specifically designated for military use, where the physicians provided additional medical care to the caliphs, military commanders, and nobility (Tschanz 2017).

### Maristan of Granada

The Maristan de Granada, one of the most famous hospitals in Al-Andalus was constructed between 1365 and 1367, is situated adjacent to the Darro River in the Axares area of Granada. It is positioned at the base of the outer walls of the Alhambra. The maristan was initially situated on a rectangular plot measuring 1015 square meters. It is thought to have been a two-story building constructed with plaster-covered bricks, following the architectural style of the Moors. The maristan featured a central courtyard with a pool supplied by a fountain. Surrounding this area were clinical living spaces, each measuring six square meters, which were interconnected by galleries. The maristan had the capacity to accommodate up to two hundred patients. The recognition of comparable Christian institutions did not occur until the fifteenth century. The edifice known as the Granada maristan underwent a transformation in the sixteenth century, evolving into the Casa de la Moneda or mint. However, by the nineteenth century, it had fallen into a state of disrepair (Garcı´a Granados, Giro´n Iruste, and Salvatierra Cuenca 1989; Hermann 1996; Torres Balba´s 1994; Almagro and Orihuela 2003). Several items from the initial years of its existence are still preserved in the adjacent Alhambra museum. There is a scarcity of documents that provide a description of the organization and operational mechanisms of the Maristan in Granada. After the successful recapture of Moorish Spain by the Catholic Kings, Christian travellers and pilgrims documented a limited number of written testimonies of the Granada Maristan (Mu¨nzer 1987).

It is worth noting that maristans in many regions of the medieval Islamic world exhibited comparable structures and purposes. In contrast to the hospices and hospitals of medieval Christianity, the maristans of medieval Islam were predominantly secular establishments. Their establishment typically stemmed from the benevolent intentions of the Sultans and relied on contributions and charitable offerings from various sources, including independent benefactors and affluent patients. Numerous maristans were characterized as educational institutions, encompassing libraries, scientific lectures, and individualized instruction, where physicians assumed a prominent position in their functioning. The clinical sections or divisions of the majority of maristans were structured based on specific ailments, with certain units specializing in the treatment of disorders, such as the care of individuals with mental illnesses, as observed at the Granada Maristan (Mu¨nzer 1987; El Ayadi 1994; Dols 1987; Elgood 1951; Cloarec 1998). Hieronymus Mu¨nzer, a renowned Austrian physician and traveller who lived approximately from 1437 to 1508, conducted a visit to the Granada Maristan in 1494. This visit took place shortly after the expulsion of Moors and Jews from Spain and Portugal. In his report, Mu¨nzer documented his views of what he referred to as a “Moorish house for lunatics” (Mu¨nzer 1987). Nevertheless, the precise purpose of the Granada Maristan as a refuge for individuals with mental illness since its establishment in 1365 remains ambiguous. However, the presence of its enduring fundamental assertions and a substantial inmate population in subsequent years indicate the possibility of a gradual transformation into a predominantly psychiatric facility (Garcı´a Granados, Giro´n Iruste, and Salvatierra Cuenca 1989; Sournia 1994).

## Influences on Later Medical and Psychological Thought

The profound impact of Andalusian contributions to mental health extended far beyond the borders of Al-Andalus. The knowledge and innovations developed by Andalusian physicians found their way into the broader Islamic world and eventually influenced European medical traditions during the Renaissance. Key Andalusian texts were translated into Latin, becoming foundational texts for the emerging field of Western medicine. Medieval physicians were influenced by bimaristans. This section discusses how Christian Europe developed medicine, how Europe embraced bimaristans’ medical successes, and how bimaristans modelled patient-centred healthcare. Muslim physicians believed diseases were caused by the body’s interaction with its environment. The basic cause and treatment of diseases were also discovered. Prophet Muhammad said, “Allah did not create an illness without a treatment. The knowledgeable are aware, while the foolish remain ignorant” (Ibn Abi Shaibah 1988). This statement may have prompted Muslim scientists to verify Muhammad’s claim and gain life-changing information. The bimaristans were not merely venues to treat patients, but they also strengthened physicians’ and patients’ spirituality by seeing ailments that were formerly deemed incurable cured (Lafuente Alca´ntara 1859).

Bimaristans are still acknowledged across Europe. The academics pavilion at Muslims, the UN office in Vienna, has monuments of Muslim physicians Rhazes (Muhammad ibn Zakariyya al-Razi, d. 925) and Avicenna. Rhazes, Avicenna, Aesclepius (c.1250 BC), and Hippocrates were among Chaucer (d. 1400), the originator of English literary tradition and the finest English poet of the Middle Ages (Abdel-Halim 2021).

Europeans in the Middle Ages valued the Muslim legacy because the bimaristans preserved European medicine, which would have lost much of European history. Latin European civilization declined after Charlemagne’s death in 814 CE. Toledo, Spain, preserved this culture (Skeat 2007). Archbishop Raymond de Sauvetât (d. 1152) of Toledo founded a translation institute to translate Arabic writings from various locales into Latin. Latin translations spread throughout Europe, especially in seventeenth-century France and Germany. Getting medical info from books was secondary. Primary medical knowledge was learned in Bimaristans, where physicians and medical students, both male and female, collaborated for instruction. Only fifty main hospitals in Cordoba addressed physical and mental diseases (Farag 1978). Western European medical institutes like Salerno, Padua, Bologna, Montpellier, and Paris emerged from this new phenomenon (Thorne 2013). In the twelfth to fourteenth century cordial interactions between the Islamic East, Andalusia, and the Latin West led to European medical institutions like the Hospital of Our Lady Mary of the Innocents in Valencia, which were bimaristans (Pérez et al 2012). The enduring legacy of Andalusian contributions is also evident in the development of psychology as a distinct discipline. The insights into the human mind and mental well-being provided by Andalusian scholars laid the groundwork for the later emergence of psychology as a scientific field, bridging the gap between philosophy and medicine.

## Ethical Significance of Andalusian Mental Health Practices

The history of Andalusian mental healthcare offers a wealth of ethical insights, particularly in relation to the treatment of vulnerable populations such as those suffering from mental illness. One of the most significant ethical dimensions of this history is the emphasis on compassionate care within the maristanes, which functioned as holistic institutions that respected the dignity of mentally ill patients. This approach stands in stark contrast to many later Western practices, where patients were often isolated in asylums with limited consideration for their humanity. The ethical principles underlying Andalusian medicine—such as the integration of spiritual, psychological, and physical care—resonate with contemporary bioethical debates about patient-centred care and the importance of addressing not only physical symptoms but also the emotional and spiritual well-being of patients.

Additionally, the ethical commitment to gender-sensitive care, as seen in the separate wards for men and women in the maristanes, reflects an early recognition of the need for privacy, respect, and culturally aligned medical care—principles that are central to modern discussions on healthcare equity and cultural competence. This history challenges the often singular narrative of Western dominance in the evolution of medical ethics, offering a pluralistic perspective that enriches our understanding of ethical practices across cultures and time periods. By exploring these dimensions, the paper provides a critical reflection on how past practices can inform current ethical frameworks in mental healthcare, making it highly relevant for a bioethical audience.

## Conclusion

In conclusion, the historical era of Islamic governance in Andalusia stands as a testament to the power of intellectual convergence and cultural exchange. This period, known as the Andalusian Renaissance, witnessed the flourishing of knowledge and the birth of ground-breaking advancements in various fields, particularly in the domain of medicine and mental health. Andalusian physicians, scholars, and philosophers made significant contributions that continue to influence our understanding of mental health and medical practice today. The holistic approach to medicine adopted by Andalusian scholars, emphasizing the interconnectedness of physical health, emotional well-being, and spiritual harmony, was far ahead of its time. Their comprehensive perspective on healthcare, which included preventive medicine, dietary advice, and psychological counselling, is a valuable lesson for modern medicine.

Andalusian medicine left an indelible mark on the world, as their knowledge and innovations were transmitted across the Islamic world and eventually into Europe. The establishment of mental health hospitals, or maristanes, in Andalusia was revolutionary, showcasing a patient-centred approach that provided holistic care for individuals suffering from mental illnesses. These institutions not only served as places of treatment but also places of spiritual and emotional healing. The impact of Andalusian contributions to mental health extended far beyond their geographical borders. Their texts were translated into Latin and became foundational texts for the development of Western medicine. The enduring legacy of Andalusian medicine is also evident in the emergence of psychology as a distinct discipline, bridging the gap between philosophy and medicine. In a world where the understanding of mental health and the treatment of mental disorders continue to evolve, the lessons from Andalusia’s intellectual heritage remain relevant and inspiring. The Andalusian Renaissance serves as a reminder of the power of diversity, cultural exchange, and the relentless pursuit of knowledge in shaping the course of human history.

Reflecting on the historical contributions of Andalusian medicine and its mental healthcare practices reveals critical ethical and philosophical insights that remain relevant today. The maristanes, as early institutions of mental healthcare, embody principles of respect, compassion, and holistic care that serve as a moral benchmark in contrast to later Western models, such as the asylum movement, which often lacked such humanity. Moreover, the Andalusian model of gender-aligned care and its attention to the spiritual and emotional dimensions of mental health underline an ethical commitment to treating the individual as a whole—a concept that still resonates in contemporary debates on patient-centred care. This history not only provides valuable insights into how mental illness was perceived and treated in a non-Western context but also challenges the predominance of Western medical narratives in bioethics. By highlighting these normative and ethical dimensions, this paper contributes to a more pluralistic and enriched understanding of mental healthcare, with lessons that are applicable to modern medical ethics, philosophy, and cross-cultural healthcare.

## Data Availability

Not applicable.
